# A New Type of Photo-Thermo Staged-Responsive Shape-Memory Polyurethanes Network

**DOI:** 10.3390/polym9070287

**Published:** 2017-07-19

**Authors:** Jinghao Yang, Hao Wen, Haitao Zhuo, Shaojun Chen, Jianfeng Ban

**Affiliations:** 1 Guangdong Research Center for Interfacial Engineering of Functional Materials, Shenzhen Key Laboratory of Polymer Science and Technology, Nanshan District Key Lab for Biopolymers and Safety Evaluation, College of Materials Science and Engineering, Shenzhen University, Shenzhen 518060, China; 2150120416yjh@gmail.com (J.Y.); 13145991523@163.com (H.W.); 2 Shenzhen Key Laboratory of Functional Polymer, College of Chemistry and Environmental Engineering, Shenzhen University, Shenzhen 518060, China; haitaozhuo@163.com

**Keywords:** azobenzene, polyurethane, shape memory, photo, responsiveness

## Abstract

In this paper, we developed a photo-thermo staged-responsive shape-memory polymer network which has a unique ability of being spontaneously photo-responsive deformable and thermo-responsive shape recovery. This new type of shape-memory polyurethane network (A-SMPUs) was successfully synthesized with 4,4-azodibenzoic acid (Azoa), hexamethylenediisocyanate (HDI) and polycaprolactone (PCL), followed by chemical cross-linking with glycerol (Gl). The structures, morphology, and shape-memory properties of A-SMPUs have been carefully investigated. The results demonstrate that the A-SMPUs form micro-phase separation structures consisting of a semi-crystallized PCL soft phase and an Azoa amorphous hard phase that could influence the crystallinity of PCL soft phases. The chemical cross-linking provided a stable network and good thermal stability to the A-SMPUs. All A-SMPUs exhibited good triple-shape-memory properties with higher than 97% shape fixity ratio and 95% shape recovery ratio. Additionally, the A-SMPUs with higher Azoa content exhibited interesting photo-thermo two-staged responsiveness. A pre-processed film with orientated Azoa structure exhibited spontaneous curling deformation upon exposing to ultraviolet (UV) light, and curling deformation is constant even under Vis light. Finally, the curling deformation can spontaneously recover to the original shape by applying a thermal stimulus. This work demonstrates new synergistically multi-responsive SMPUs that will have many applications in smart science and technology.

## 1. Introduction

Shape-memory polymers (SMPs) have attracted significant attention due to their potential application in various areas, such as biomedical devices [1,2], smart textiles [3] and engineering [4]. Shape-memory polymers can adopt temporary shapes and spontaneously transform to their original shapes when exposed to a specific external stimulus, such as heat, light, electric, and chemical moieties [5,6]. Based on the different stimulus, SMPs can be systematically divided into thermo-responsive [7], photo-responsive [8], electro-responsive, chemo-responsive, and so on [6]. Most of the existing SMPs are thermo-responsive, and they exhibit strictly thermal-dependent performance, which are greatly hindered by the steps of heat-demanding programming and thermal-triggered recovery. Recently, light actuation has received more attention [9,10]. Photo-responsive SMPs contain a light-switching function element that undergoes isomerization when exposed to irradiation with a specific wavelength of light [8]. Compared with thermo-responsive, photo-responsive have many more advantages, such as remote-responsiveness and green environmental stimulus, whereas, the thermos-responsiveness cannot satisfy complex needs from practical applications. Thus, researchers are encouraged to actively develop multi-responsive SMPs and multi-functional SMPs [11,12,13,14,15].

Recently, multi-responsive SMPs have drawn the attention of many researchers [11,12]. What makes the multi-responsive SMPs more interesting and appealing is the combination of several stimuli and unique properties. Most of them show dual-responsive shape-memory effects (SMEs), such as thermo/solvent dual-responsive SMEs, thermo/water dual-responsive SMEs, and thermo/photo dual-responsive SMEs [16,17,18,19,20,21,22]. There are also triple-responsive SMPs, such as thermo-, photo- and chemo-responsive SMPs [23,24]. In these multi-responsive SMPs, polymer chains usually undergo reversible alteration in structure and morphology upon exposure to light, heat and chemicals. The significant transformation of shape is associated with monomeric units’ isomerization or phase transitions. These unique properties make shape-memory polymeric materials attractive and useful candidates for the many applications in the field of flexible and intelligent actuators, nanomedicine and drug delivery. However, most of the present multi-responsive SMPs show only independent stimulus-responsiveness. The reported dual-responsive or triple-responsive SMEs were generally as a result from the simple addition of several different stimuli-responsive materials. Most recently, Xie et al. proposed a new strategy to achieve thermo- and photo-responsive triple-shape memory with non-overlapping effects in one programming cycle [25]. That study will encourage the researchers herein to design new multi-responsive SMPs [26,27]. Particularly, the shape-memory polyurethanes (SMPUs) are more attractive since they have the advantages of good mechanical properties, adjustable structure and good shape-memory properties [28,29].

**Figure 1:**
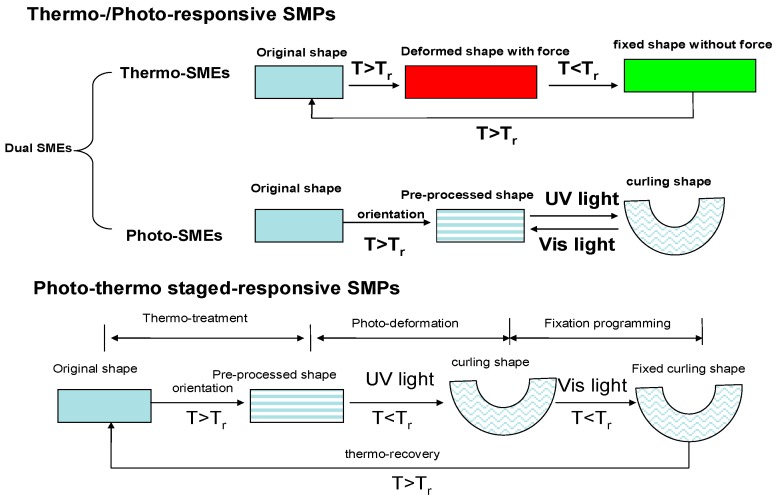
Illustration for the photo-thermo staged-responsive shape-memory polymers (SMPs) compared to the thermo/photo dual-responsive SMPs. (T_r_ is the shape recovery temperature, the traditional thermo-/photo-responsive SMPs show dual-responsive shape-memory effects (SMEs), having thermo-responsive and photo-responsive SME, respectively. However, the photo-thermo staged-responsive SMPs contain several synergistic stimulus-responsive shape-memory imparting moieties such as to provide thermo-treatment, photo-deformation, fixation and thermo-recovery).

Herein, we have developed a multi-responsive SMPs showing photo- and thermal-responsiveness that can be applied over two stages [27]. Being quite different to those of photo-/thermo dual-responsive SMPs, the photo-thermo two-staged responsive SMPs show spontaneous shape deformation by using a photo-stimulus like UV light, and can recover to the original shape by applying a thermal stimulus (Figure 1). In this communication, a new azobenzene shape-memory polyurethane network (A-SMPU) with photo-thermo two-staged responsiveness was synthesized by incorporation of polycaprolactone (PCL) soft segments and azobenzene segments serving and allowing for heat-switches and light-switches, respectively. Aiming at achieving good shape recovery, chemical-crosslinking was formed partly by using glycerol. This paper presents insights into the structure, morphology and properties of the target A-SMPUs. In particular, triple-shape-memory properties and photo-thermo two-staged responsive shape-memory properties are studied.

## 2. Experimental

### 2.1. Materials

Hexamethylenediisocyanate (HDI), 4-aminobenzoic acid, acetic acid, sodium hydroxide, glucose, lauryl two lauryl laurate and glycerol (Gl) which all are analytical grade, and *N*,*N*-Dimethylformamide (DMF, high-performance liquid chromatography grade) were purchased from Aladdin Chemical Reagent Co., Ltd. (Shanghai, China). Extra-pure grade polycaprolactone with number-average molecular weight of 4000 (PCL4000), was dried beforehand at 80 °C under 0.1–0.2 MPa for 3 h. The other chemicals were used without further purification.

### 2.2. Preparation of Azobenzene Shape-Memory Polyurethanes (A-SMPUs)

**Figure 2:**
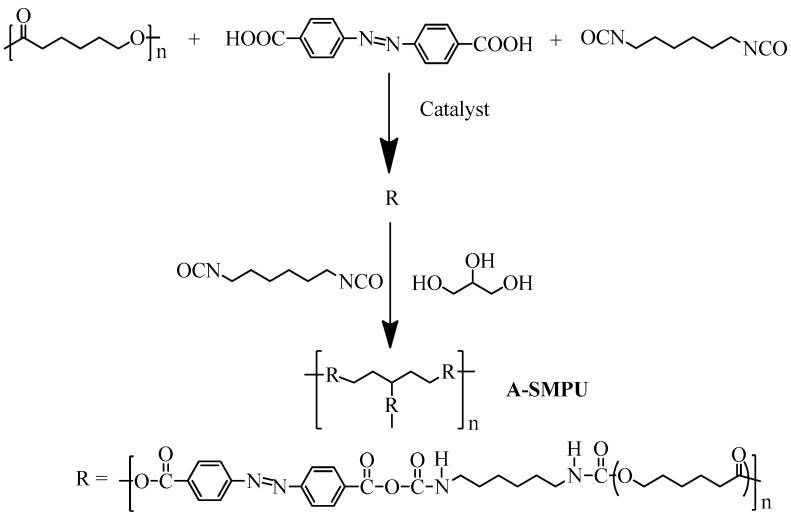
Synthesis route of the azobenzene shape-memory polyurethanes (A-SMPUs).

4,4-azodibenzoic acid (Azoa) was synthesized in our lab (see supporting information Figure S1). The A-SMPUs were then synthesized via a solution polyurethane polymerization method from Azoa, HDI, PCL4000 and Gl. The synthesis route of the A-SMPUs is presented in Figure 2, and the compositions of each sample are summarized in Table 1. The detailed procedure was carried out as follows (taking P4 as an example): Azoa (0.65 g, 20.10 mmol), polycaprolactone (PCL4000, 14.35 g, 35.90 mmol), hexamethylenediisocyanate (HDI 4.0 g, 23.80 mmol), 0.01 g lauryl two lauryl laurate serving as the catalyst, 2 mL DMF were added to a 250 mL conical flask equipped with a mechanical stirrer, and the pre-polymer was constantly stirred at 80 °C for 20 min. HDI (0.6 g, 35.70 mmol) was added to the reaction for another 2 h for chain extension. Thirdly, 10 wt % Glycerol/DMF solutions containing 0.37 g G1ycerol (Gl) were added to the reaction system within 30 min. After reaction for 3 h, the resulting polymer was poured into a Teflon pan for a post-curing process at 80 °C under a blast oven for 5 h. Finally, the target A-SMPU resin was obtained. In this paper, the samples of A-SMPUs were coded as P1, P2, P3 and P4.

### 2.3. Characterizations

Fourier transform infrared spectroscopy (FT-IR) spectra were scanned from smooth 0.2 mm thick polymer films using a Nicolet 760 FT-IR spectrometer (Nicolet, Madison, WI, USA) by using FT-IR attenuated total reflection (ATR) method. Ten scans at a resolution of 4 cm^−1^ were signal-averaged and stored for further analysis.

The X-ray photoelectron spectrometer analysis (XPS, VG multilab2000, Thermo Electron Corporation, Waltham, MA, USA) was carried out using anode voltage and current of 15 kV and 10 mA, respectively. The elemental composition was determined on the basis of peak areas and sensitivity factor from C_1s_, N_1s_, O_1s_ and S_2p_ peaks using Advantage software. All binding energy values were confirmed with reference to carbon (C_1s_ = 284.6 eV).

Nanonavi E-Sweep (SII Nanotechnology Inc., Shanghai, China) atomic force microscopy (AFM) was used in tapping mode for morphological characterization of the dried sample. The samples were dissolved in DMF at a concentration of 5 mg/mL and spin-coated firstly at 400 rpm for 10 s, and then at 4000 rpm for 60 s on oxidized silicon substrates. Spin-coated films were kept in a 50 °C oven for 48 h to evaporate the solvent.

Wide-angle X-ray diffraction (WAXD) measurements were obtained using a D8 Advance (Bruker, Billerica, Germany) instrument with an X-ray wavelength of 0.154 nm at a scanning rate of 12°/min. Specimens 0.5 mm thick were prepared for these measurements.

The morphology of the samples was examined using scanning electron microscopy (SEM, Hitachi, Hitachi, Japan). Prior to scanning, the samples were coated with a thin layer of gold.

Differential scanning calorimetry (DSC) testing was performed using a TA Q200 instrument (TA instrument, New Castle, DE, USA) with nitrogen as the purged gas. Indium and zinc standards were used for calibration. Samples were firstly heated from −60 to 200 °C at a heating rate of 10 °C/min and kept at 200 °C for 1 min, subsequently, cooled to −60 °C at a cooling rate of 10 °C/min, and finally heated a second time from −60 to 200 °C.

Thermogravimetry Analysis (TGA) curves were recorded on a computer-controlled TG Q50 system (TA instrument), under the following operational conditions: A heating rate of 10 °C/min, a temperature range of 100–600 °C, a sample weight of approximately 5.0 mg, using film samples in platinum crucibles, and 60 mL/min N_2_ flow. Three or four repeated readings (temperature and weight loss) were made for the same thermogravimetric (TG) curve and Differential thermal gravity (DTG) curves, each including to at least 15 points.

The shape-memory behaviors were determined by thermo-mechanical analysis using a TA Instruments DMA800 (TA instrument) (using tension clamps in controlled force mode) according to the procedure described in Ref. [26].

For triple-shape-memory cycles: (1) Heating to 100 °C and equilibrating for 20 min; (2) a rectangle specimen with 20 mm × 5 mm × 0.1 mm was uniaxially stretched by ramping force from 0.001 to 1 N at a rate of 0.25 N min^−1^; equilibration for 3 min; (3) fixing the strain by rapid cooling to 45 °C at −10 °C/min, followed by equilibration for 10 min; (4) further fixing the strain by rapid cooling to 0 °C at −10 °C/min, followed by equilibrating for 10 min; (5) unloading external force 0 N at a rate of 0.25 N min^−1^; (6) reheating to 45 °C at a rate of 4 °C/min and equilibrating for 40 min; (7) reheating to 100 °C at a rate of 4 °C/min followed by equilibration for 40 min.

For photo-thermo staged-responsiveness: (1) A rectangle specimen with 20 mm × 5 mm × 0.1 mm was cut from the test sample; (2) the specimen was stretched to about 240% strain, with rapid cooling to room temperature; (3) the fixed specimen was further exposed to UV light (365 nm) for around 10 s; (4) the specimen was equilibrated to room temperature and exposed to visible light; (5) the specimen was further heated above 50 °C for 20 min. The shape-changing during the programming was recorded for analysis.

## 3. Results and Discussion

### 3.1. Molecular Structure Analysis

**Figure 3:**
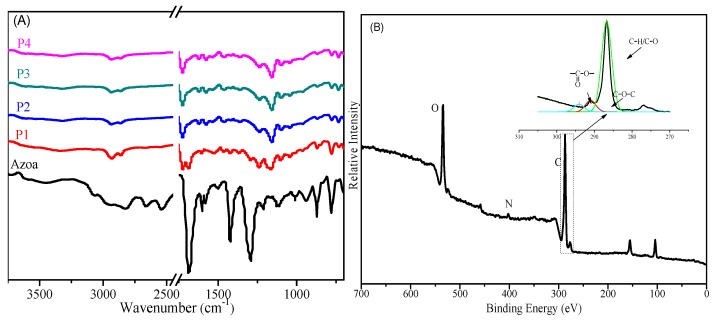
Spectra of A-SMPUs ((**A**)-FT-IR spectra, (**B**)-XPS spectra of the sample P1).

FT-IR was employed to confirm the chemical structure of the A-SMPU. Figure 3A shows the FT-IR spectra of Azoa and the A-SMPU. In all samples of A-SMPU, there were no COOH absorption peaks at approximately 2500–3500 cm^−1^, whereas C=O and N=N stretching vibrations of Azoa were observed at approximately 1610 cm^−1^ and 1420 cm^−1^ [27]. These results indicated that the Azoa had reacted with HDI, forming hard segments completely. In addition, the N–H stretching vibration and C=O stretching vibration were detected at approximately 3328 and 1725 cm^−1^, respectively, suggesting the existence of strong hydrogen bonding between the N–H and C=O groups in urethane. Additionally, no N=C=O characteristic absorption peak was observed at approximately 2260 cm^−1^, confirming the successful formation of polyurethanes. These results thus suggest that the target A-SMPUs were successfully prepared in this experiment. To further evaluate whether A-SMPUs were cross-linked chemically with Gl, XPS was used to determine the main elements and the carbon-based bonds of this polymers network. As shown in Figure 3B, the low-resolution spectra of all of the P1 showed that carbon and oxygen atoms were the main components, whereas less nitrogen and sulfur were also present. In the high-resolution carbon spectra, the carbon signal could be resolved into several component peaks, which reflected the local environments of the carbon atoms (C–C and C–H or C–O–C or O–C=O). These results indicated that the hydroxyl groups of Gl successfully reacted with HDI. Moreover, the A-SMPUs are less dissolved by any solvents like DMF. Therefore, the target A-SMPUs contain chemical-crosslinks.

### 3.2. Morphology Analysis

**Figure 4:**
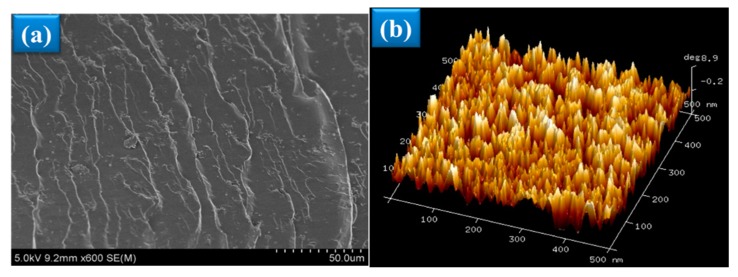

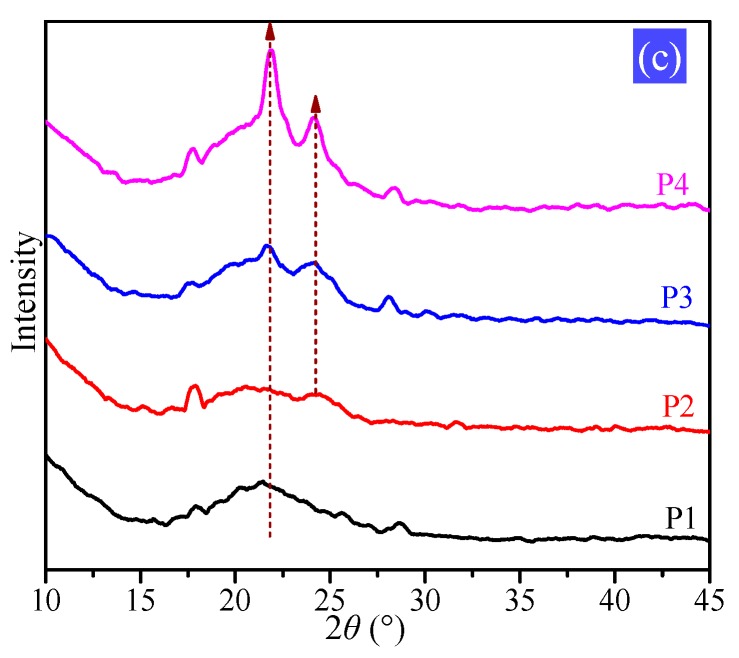
Morphology analysis of A-SMPUs. (**a**) Typical SEM image; (**b**) typical atomic force microscopy (AFM) images; (**c**) wide-angle X-ray diffraction (WAXD) patterns of the A-SMPU.

The surface morphology of the A-SMPUs films was investigated systematically with SEM and AFM. The SEM images show that all polymers exhibit a more uniform crack on the surfaces, due to brittle fracture behavior. When Azoa content decreased, the quantity of the cracks decreased, and the fracture direction became less uniform (Figure S3). The sample P4 exhibited a simple crack on the surface without lamination, while more cracks and lamination appeared on the surfaces of sample P1 (Figure 4a). It was confirmed that the rigidity of polymer increased with an increase of Azoa content since the higher content of rigid Azoa structures were incorporated into the polyurethane serving as hard segments. AFM images further confirmed the formation of a micro-phase structure composed of hard phase and soft phases in the polyurethane. Figure 4b demonstrates that raised and concave sections were evenly distributed across the surface. The light-colored regions are ascribed to the hard segments of A-SMPUs while the concave regions belong to the soft segments. In addition, comparing with sample P4, the raised peaks were more intense and broad in sample P1 (Figure S4), which illustrates higher content of hard domains.

This micro-phase separated structure provides a morphological foundation that exhibited good shape-memory properties. The micro-phase separation morphologies of the A-SMPUs were further characterized using wide-angle X-ray diffraction (WAXD). Figure 4c demonstrates that the A-SMPUs have crystallinity changes according to varying Azoa content. Sample P1 shows a broad and disperse diffraction peak located in the wide-angle region, 2θ = 10~35°, and indicates an amorphous morphology. As the Azoa content decreases, crystalline peaks appear in sample P2. The intensity of crystalline peaks increases with the decrease of Azoa content. Finally, two clear crystalline peaks are observed at 21.8° and 24.2° in sample P4, which were indexed as the (110), (200) planes, ascribing to the PCL crystals. These results indicate that the A-SMPUs can form semi-crystalline soft phases and amorphous hard phases. The soft phase was influenced by the hard segments structure, and the increased Azoa structure disrupts the crystallinity of the soft phase. These influences may also be reflected in the thermal properties and phase transitions.

### 3.3. Thermal Properties of the A-SMPUs

**Figure 5:**
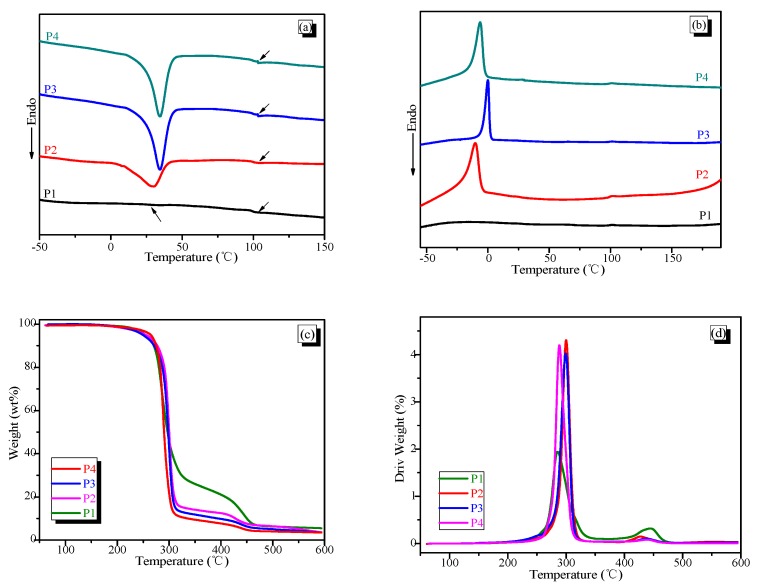
Thermal-properties of A-SMPUs: (**a**) The second differential scanning calorimetry (DSC) heating curves; (**b**) the DSC cooling curves; (**c**) the thermogravimetric (TG) curves; (**d**) DTG curves.

Differential scanning calorimetry (DSC) has been widely used to study the phase transitions of polymers. Figure 5a,b presents the DSC curves of A-SMPUs. In being consistent with the previous XRD results, sample P1 shows no crystal melting transitions since there are no PCL crystals. The crystal melting temperature (*T*_m_) of PCL soft phase occurs in sample P2, P3 and P4 while a weak glass transition occurs at about 100 °C. Accordingly, the crystallization temperatures were detected on the cooling curves (Figure 5b). As the PCL soft-phase content increases, the peak area for crystal melting increases linearly from P2 to P4, confirming the increasing crystallinity. Additionally, comparing with the pure PCL- or the common PCL-based polyurethanes, the crystallinity is much lower for the A-SMPUs. The reason is that the crystallinity of PCL was inhibited by the hard segments, cross-linking, and being a small fraction of the overall polymer. In addition, the *T*_m_ of the PCL soft phase also shifted to the lower temperature as the Azoa content increased because the molecular chain of the soft segments was limited by the rigid Azoa structure. In contrast, the glass transition temperature (*T*_g_) of hard segments tends to shift to the higher temperature as the Azoa content increases. It is further confirmed that the Azoa structure can promote high thermal performance and increased amorphous behavior.

The thermal properties of A-SMPUs were also investigated with thermogravimetric analysis (TG) (Figure 5c,d). The TG curves show that the initial decomposition temperature of A-SMPUs are higher than 270 °C and indicates good thermal stability (Table S1). As the temperature increases, a second decomposition stage occurs at 425 °C. The weight loss of the second decomposition increases with increasing Azoa content. It is thus confirmed that the PCL soft segment is decomposing on the first stage at about 270 °C, while the second decomposition stage is attributed to the hard segments. Derivative TG curves further show that the second decomposition temperature shifts to lower temperature range as the Azoa content decreases. The possible reason is that the rigid Azoa can promote the aggregation of the hard segments, as discussed in SEM and AFM results. Therefore, these results confirm that the A-SMPUs have good micro-phase separation structures consisting of PCL-based semi-crystalline soft phases and Azoa-based amorphous hard phases. These structures are expected to show good thermo-responsive SMEs.

### 3.4. Triple-Shape Memory Properties

**Figure 6:**
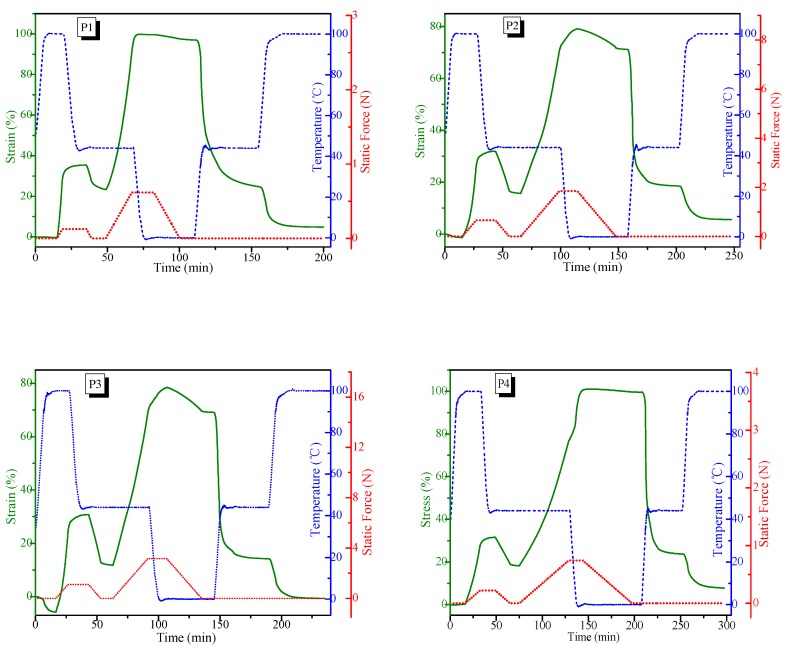
Triple-shape-memory behavior of the A-SMPUs.

Thermo-responsive shape-memory properties of the A-SMPUs were investigated using dynamic mechanical analysis. The strain recovery process and its dependence on the temperature change were recorded for analysis. Figure 6 presents the whole strain-time-temperature curves of samples P1, P2, P3 and P4, respectively. The results demonstrate that the samples can fix two temporary shapes and recover them under the program of two-staged thermo-responsive shape recovery, showing the typical triple-shape-memory effects [26]. Quantitative analysis shows that the total strain recovery ratio were above 92% in all samples (Table S2), because the chemical cross-linked structure imparts good elasticity rubbers upon heating. Taking P1 as an example, the second strain fixity ratio is 97% at 0 °C. During the first recovery process, sample P1 recovered 65.9% of its strain at 45 °C, and P1 recovered the remaining 29% of strain when the temperature was increased to 100 °C. Finally, more than 95% of the total strain was recovered. Generally, the first shape fixity ratio was lower than the second shape fixity ratio in all samples, particularly the sample with the low Azoa content. The reason is that the first shape fixation should result from the glass transition of hard phase, while the second shape fixation results from the crystallization of the soft phase. The chemical cross-linking provides the stable crosslink points for strain recovery.

### 3.5. Photo-Thermo Two-Staged Responsive Behaviors

**Figure 7:**
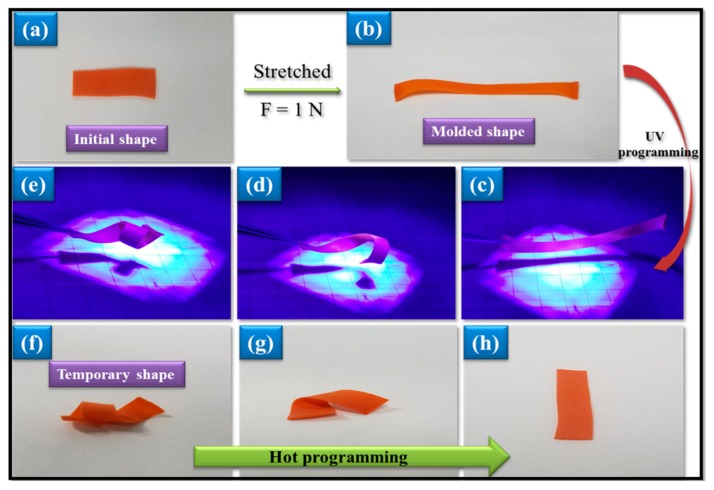
Photo-thermo staged-responsive shape-memory behaviors (The original shape (**a**) was first thermo-treated to a flattened shape (**b**). By applying UV light stimulus, the flattened shape was spontaneously transformed into curling shapes (**c**–**e**) even at low temperature, and the curling shape was kept constant under visible light at low temperature. When a thermo-stimulus is applied, the thermo-recovery occurs and the curling shape was transformed into the original shapes (**f**–**h**)).

Based on the structure analysis above, the crystal melting transition of PCL soft phase is thermo-responsive, while the Azoa structure could show photo-responsive behavior since the Azoa structure shows cis-trans isomerization about the N=N bond from trans to cis. Thus, the combination of thermo-responsiveness and photo-responsiveness was investigated. First, it was found that all A-SMPUs can be elongated to a high deformation above 50 °C, and the temporary deformation can be fixed at room temperature. Similar to the triple-SMEs above, these temporary deformations recovered quickly by heating to above 50 °C. These are typical thermo-responsive behaviors, but when the A-SMPUs were exposed to the UV light, no deformation occurred because of the low Azoa content. After the original film was stretched at higher temperature and followed by fixing at lower temperature, the orientation of Azoa segments is thus formed and a temporary shape was obtained (Figure 7a,b). By applying UV light, the molded film was found to curl gradually upon exposure to the UV light (Figure 7c–e), this is a stage of spontaneous photo-responsive shape deformation. Interestingly, the curling shape does not recover after ceasing the UV, and even exposing to visible light. This interesting behavior is quite different from the common photo-responsive SMPs (Figure 1), whereas the deformed curl shape can be recovered quickly to its original shape or length with hot programming by heating above 50 °C (Figure 7f–h). This stage should belong to the thermo-responsive shape recovery. Therefore, the A-SMPUs exhibit a novel two-staged responsiveness, consisting of spontaneous photo-responsive shape deformation and spontaneous thermo-responsive shape recovery.

Considering the necessary requirements for photo-thermo two-staged responsiveness, pre-orientation of polymer chains and higher content of Azoa content are required in the cross-linked A-SMPUs. According to the described structure and morphology analysis, the mechanism for this behavior is explained as follows: Through the first thermal-treatment process, Azoa molecules form a highly orientated structure and the temporary orientation is fixed by the crystallization of the PCL soft phase and the hydrogen bonding between hard segments. Upon exposure to the UV irradiation, the Azoa structure changes from *trans*-isomers to *cis*-isomers, resulting in wave deformation of the disorientation occurring quickly on the upper surface of the film. Therefore, spontaneous photo-responsive deformation is achieved on the first stage. After ceasing UV exposure, the Azoa *cis*-isomers are fixed by strong intermolecular forces and the PCL crystalline soft phase. Therefore, no deformation occurs under visible light. However, when the temperature increases to the *T*_m_ of soft phase, melting of PCL crystals destroys the hydrogen bonding of hard segments and the intermolecular forces of polymer chains weaken. Therefore, the temporary shape could be recovered upon heating by the elasticity of polymer networks. This mechanism has been verified in our previous communication [27].

## 4. Conclusions

This work presents a new type of photo-thermo responsive shape-memory polyurethane that is capable of spontaneous photo-deformation and thermal shape recovery. The azobenzene (Azoa)-containing polyurethane with different Azoa-content was successfully prepared with PCL4000, Azoa, hexamethylenediisocyante and glycerol. The effect of Azoa content on the structure, morphology and properties, particularly triple-shape memory and the photo-thermo-responsiveness was carefully investigated. The results demonstrate that the chemical cross-linked A-SMPUs form micro-phase-separated structures consisting of a semi-crystalline PCL4000 soft phase and amorphous Azoa-based hard segments. The increased Azoa content or hard segment content influences the crystallinity of the PCL soft phase. All A-SMPUs exhibit good triple-shape-memory properties with higher than 97% shape fixity ratio and 95% shape recovery ratio. The A-SMPUs with higher Azoa content also exhibits photo-thermo two-staged responsiveness. Further, a synergistic multi-responsive shape-memory polymer was successfully formed and is expected to have many applications in the development of smart materials, robotics, and smart sensors.

## Figures and Tables

**Table 1:** Composition of the azobenzene shape-memory polyurethanes (A-SMPUs).

Samples	Hard segments		Soft segments		Gl (g)	Content of azoa (wt %)
	Azoa (g)	HDI (g)	PCL (g)	HDI (g)		
P1 (8:2)	3.20	1.98	11.80	0.50	0.90	17.4
P2 (6:4)	1.40	0.85	13.60	0.57	0.52	8.2
P3 (5:5)	0.95	0.59	14.05	0.59	0.43	5.7
P4 (4:6)	0.65	0.40	14.35	0.60	0.37	4.0

## Supplementary Materials

The following are available online at www.mdpi.com/2073-4360/9/7/287/s1.
