# Supplementary material for: A New Type of Photo-Thermo Staged-Responsive Shape-Memory Polyurethanes Network
Source: Polymers (Basel). 2017 Jul 19;9(7):287. doi: 10.3390/polym9070287 (PMC6431991; doi:10.3390/polym9070287)
Supplement: Supplementary file 1 [file polymers-09-00287-s001.pdf]

polymers

MDPI

Supplementary Materials

Experimental section

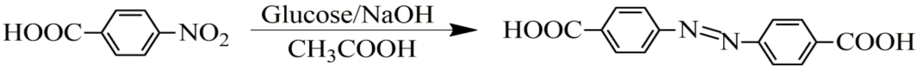

Azoa

Figure S1. Synthesis route of the 4,4-azodibenzoic acid.

4,4-azodibenzoic acid (Azoa) was easy to synthetic According to our previous work. The chemical structures and synthetic procedures for the Azoa are shown in scheme S1. The characterization data of the monomer were confirmed by ^{1}H-NMR and Mass Spectrometry (MS).The characterization data of the monomer are as follows: Azoa (Fig. S1), ^{1}H NMR (δ, ppm, DMSO-d$_6$): 12.23 (a, 1H, -COOH), 8.47-7.71 (b, 4H, Ar-H), 6.71 (c, 4H, Ar-H). ppm = 3.33 and 2.67, which belong to the solvent of DMSO-d6. Mass spectrometry (MS) (m/z) [M] calcd for C14H10N2O4, 270.24; found, 270 + 1.

Supplementary tables and figures

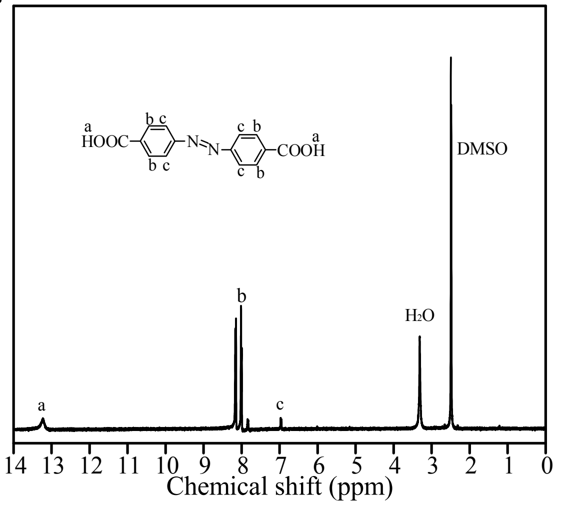

Figure S2. ^{1}H-NMR spectra of Azoba in DMSO-d$_6$.

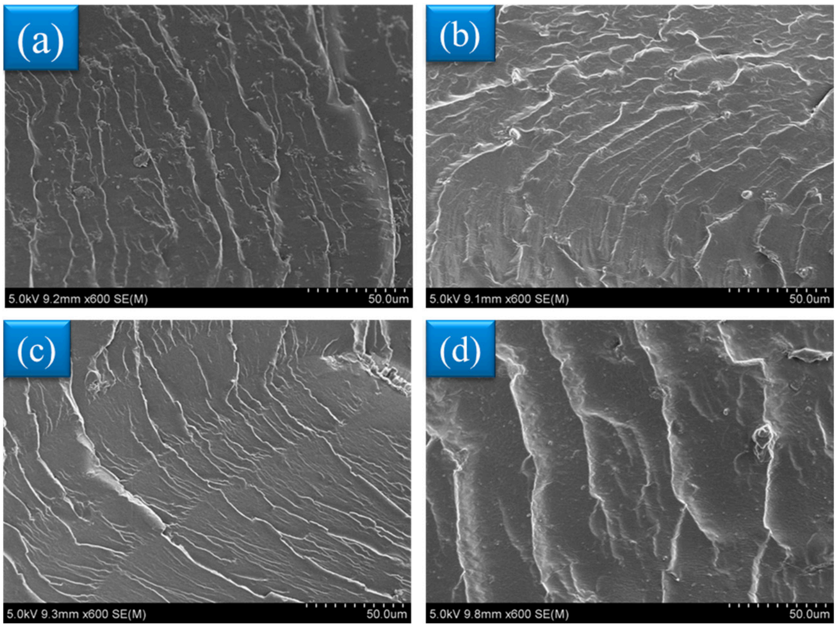

Figure S3. SEM images of (a) P1, (b) P2, (c) P3, and (d) P4, respectively.

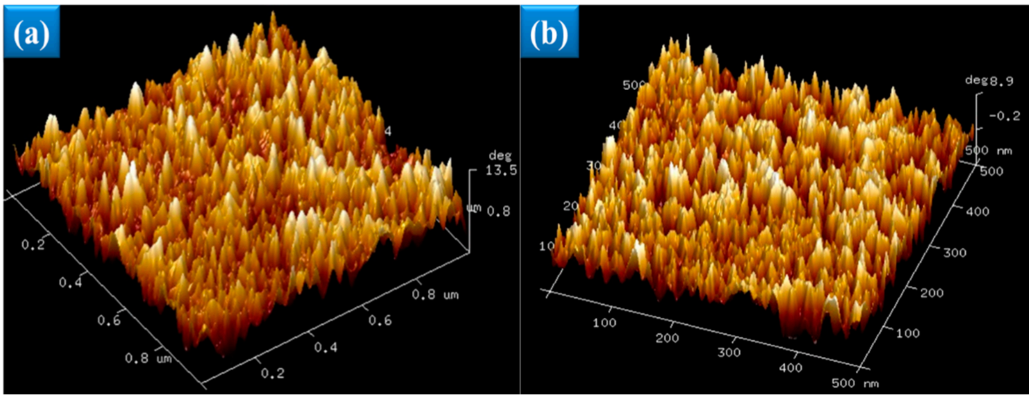

Figure S4. Typical AFM images of (a) P1, and (b) P4, respectively.

Table S1 Thermal properties of the A-SMPUs

Sample	T_{d1} (°C)[a]	T_{d2} (°C)[a]	T_m(°C)[b]	T_g(°C)[b]
P1	204.3	382.8	25.1	104.3
P2	212.6	397.6	30.3	103.5
P3	221.0	410.9	34.2	103.1
P4	234.2	410.4	33.6	102.9

a. T_{d1} is the peak temperature on the first stage; T_{d2} is the peak decomposition temperature on the second stages, measured by DTG.
b. Evaluated by DSC during the second heating process at a rate of 10 °C min^{-1} under nitrogen atmosphere.

Table S2 Shape recovery ratio and shape fixity ratio of A-SMPUs in the triple shape memory cycle

Samples	First shape fixity ratio ratio (%)	Second shape fixity ratio ratio (%)	First strain recovery ratio (%)	Second strain recovery ratio (%)	Total strain recovery ratio (%)
P1	65.9	96.1	98.0	80.6	95.2
P2	49.4	87.7	94.8	70.0	94.4
P3	38.7	86.3	95.9	96.3	96.1
P4	57.9	98.3	93.3	67.2	92.2
